# *In Vivo* Efficacy of a Synthetic Coumarin Derivative in a Murine Model of Aspergillosis

**DOI:** 10.1371/journal.pone.0103039

**Published:** 2014-08-20

**Authors:** Seema Singh, Rajesh Dabur, Madhumanjiri M. Gatne, Bharat Singh, Shilpi Gupta, Sharad Pawar, Sunil K. Sharma, Gainda L. Sharma

**Affiliations:** 1 Diagnostic Biochemistry, CSIR-Institute of Genomics and Integrative Biology, Delhi, India; 2 Department of Biotechnology, University of Pune, Pune, India; 3 Department of Biochemistry, Maharishi Dayanand University, Rohtak, India; 4 Department of Pharmacology and Toxicology, Bombay Veterinary College, Parel, Mumbai, India; 5 Department of Chemistry, University of Delhi, New Delhi, India; 6 Department of Pharmacology, National Research Institute of Basic Ayurvedic Sciences, Kothrud, Pune, India; University of Wisconsin Medical School, United States of America

## Abstract

Despite advances in therapeutic modalities, aspergillosis remains a leading cause of mortality. This has necessitated the identification of effective and safe antifungal molecules. In the present study, *in vivo* safety and antifungal efficacy of a coumarin derivative, *N*, *N, N*-Triethyl-11-(4-methyl-2-oxo-2*H*-benzopyran-7-yloxy)-11-oxoundecan-1-aminium bromide (SCD-1), was investigated. The maximum tolerable dose of compound was determined according to OECD 423 guidelines. The compound could be assigned to category IV of the Globally Harmonized System and its LD_50_ cut-off was found to be 2000 mg/kg body weight. The survival increased in *Aspergillus fumigatus-*infected mice treated with a dose of 200 mg/kg, orally or 100 mg/kg body weight, intraperitoneally, of SCD-1 in comparison to infected-untreated animals. The SCD-1 treatment resulted in significant reduction in colony counts in vital organs of the animals. Its protective effect was also observed on day 14 as there was marked reduction in fungal colonies. The treatment with SCD-1 also reduced the levels of serum biochemical parameters with respect to infected-untreated animals. It could be concluded that SCD-1 is a quite safe antifungal compound, which conferred dose dependent protection against experimental aspergillosis. Therefore, SCD-1 holds potential for developing new formulations for aspergillosis.

## Introduction

*Aspergillus fumigatus* is an opportunistic filamentous mould responsible for more than 90% of infections caused due to *Aspergillus* [1]. The clinical manifestations of diseases caused by *A. fumigatus* are varied and may range from allergic, semi-invasive to invasive conditions, depending upon immune status of the host [2]. The invasive aspergillosis (IA) is mostly fatal and the mortality rate due to IA has been observed to vary from 50–95% [3]–[5] and reaches upto 100%, when the disease is not treated or has become disseminated to vital organs [6], [7].

The drugs used to treat IA are limited in number and their therapeutic utility is hampered by side effects and development of resistance in the pathogen [8], [9]. Despite several side effects, Amphotericin B has been the mainstay of aspergillosis therapy since last many decades. The lipid formulations of Amphotericin B have also been developed to overcome its dose-dependent toxicity but although less toxic, they are expensive [10] and also exhibit variability in their pharmacokinetics, tissue distribution and safety levels [11], [12]. Another class of antifungal agents is azoles, among them, voriconazole has been used for the primary therapy for IA, with posaconazole and itraconazole as the drugs for salvage therapy [13]. Similar to other antifungals, there have been increased incidences of resistance to azole antifungals in *A. fumigatus* [14], [15]. The echinocandins have been most promising but are available only as parenteral formulations [16].

Despite the continuous advancements in the field of antifungal drug discovery, the mortality rate due to IA remains high [17], [18]. Therefore, the development of an ideal antifungal drug remains challenging and elusive. This has driven the work on discovering novel and effective antifungal molecules, from natural and synthetic sources, which will target pathway(s) different than those targeted by current drugs to exert specific activity with minimal or no side effects.

The molecules belonging to class of coumarins have attracted considerable interest in recent years, owing to their diverse pharmacological properties [19]–[22]. The investigations on synthetic coumarin derivatives (SCDs) led us to identify an antifungal molecule *N*, *N*, *N*-Triethyl-11-(4-methyl-2-oxo-2*H*-benzopyran-7-yloxy)-11-oxoundecan-1-aminium bromide coded as SCD-1 [23]. The compound exhibited potent activity against pathogenic *Aspergilli* (MIC_90_ 15.62 µg/mL) and the possible mechanism of action of SCD-1 was described using differential proteomics in our earlier report [24]. The treatment of *A. fumigatus* with SCD-1 resulted in complete inhibition of proteins belonging to key metabolic pathways of cell replication and also the riboflavin biosynthesis, which has been a pathogen-specific process [24]. The present study deals with evaluation of safety and antifungal efficacy of SCD-1 by using experimental animals.

## Materials and Methods

### Coumarin

The coumarin used in the present study was a synthetic coumarin derivative (SCD-1), a well characterized quaternary ammonium alkyl ester [23] with chemical formula *N, N, N*-triethyl- 11-(4-methyl-2-oxo-2*H*-chromen-7-yloxy)-11-oxoundecan-1-aminium bromide (molecular weight 444.3114). It was synthesized in laboratory according to the procedure described in our earlier report [23]. In view of the promising *in vitro* antifungal activity, safety and significant impact on the proteomic machinery of *A. fumigatus* [24] the compound was considered to be an important candidate molecule, therefore, investigated for *in vivo* safety and antifungal efficacy in this study.

### *In vivo* toxicity

The acute oral toxicity of SCD-1 was determined according to the Organization for Economic Co-operation and Development (OECD) guidelines 423 [25]. The mice were maintained at animal house, Bombay Veterinary College, Mumbai, India. The experimental protocol was in accordance with guidelines of Committee for the Purpose of Control and Supervision of Experimental Animals (CPCSEA) and approved by the Institutional Animal Ethics Committee (approval No. MVC/IAEC/08/2011), Bombay Veterinary College, Mumbai, India. The experiments were conducted on healthy female swiss albino mice of 8–10 weeks, obtained from the animal house, Bombay Veterinary College, Mumbai, India. The temperature of the animal house was maintained at 25°C with 30–70% relative humidity and controlled 12 h light: dark cycle. Mice were acclimatized to laboratory conditions for 5 days before initiation of the study. Animals were housed in properly labeled polypropylene cages and fed on standard diet and potable water *ad libitum*. They were kept on fasting for 3–4 h prior to treatment with the compound. Predefined doses of 2000, 300, 50 or 5 mg/kg body weight (bw) of SCD-1 were given sequentially by the oral route. The animals were weighed daily and observed for any change in the behavior, symptoms of toxicity and mortality, if any, over a period of 14 days. The animals were euthanized by CO_2_ asphyxiation followed by cervical dislocation at the end of experiments for post-mortem observations.

### *In vivo* antifungal efficacy

#### Pathogen

The *A. fumigatus* strain used in the present study was a clinical isolate obtained from patient of allergic bronchopulmonary aspergillosis and characterized at Vallabhbhai Patel Chest Institute, Delhi, India. It was further typed at Indian Type Culture Collection (ITCC6604), Indian Agriculture Research Institute, New Delhi, India.

#### Preparation of inoculum

The *A. fumigatus* culture was maintained on Sabouraud dextrose agar (SDA) plates (Himedia, Mumbai, India) in a biological oxygen demand incubator (Calton, NSW, India) at 37°C. The conidia were harvested in phosphate buffered saline (PBS) containing Tween-80 (0.1%) and scraped with a sterile disposable cotton swab followed by filtration through two layers of sterile gauze to remove fungal hyphae. Tween-80 was completely removed from conidial suspension by washing with PBS and finally the conidia were suspended in same buffer. The conidia were counted using a hemocytometer and adjusted to 1×10^8^ conidia/ml in the inoculum.

#### Immunosuppression

The animals were subjected to immunosuppression using a combination of cortisone acetate and cyclophosphamide [26]. Two days prior to infection with *A. fumigatus* conidia, the cortisone acetate (250 mg/kg bw) was given subcutaneously to animals, while a similar dose (250 mg/kg bw) of cyclophosphamide was administered intraperitoneally. Another shot of immunosuppression was given on 3^rd^ day post infection with same dose of cortisone acetate i.e. 250 mg/kg bw whereas dose of cyclophosphamide was reduced to 200 mg/kg bw. All standard animal husbandry practices were followed meticulously during the course of study. Appropriate steps were adopted to keep the mice free from stress or discomfort. To further prevent distress to animals, humane endpoints were established at the very beginning of experiment. The daily feed and water intake (data not shown), was recorded in addition to the measurement of body weight of the animals. Throughout study, the mice were examined 3–4 times daily for clinical signs such as rapid or very slow, shallow and labored breathing, ruffled fur, hunched posture, impaired ambulation, lethargy/drowsiness, aversion to activity and ulcerative dermatitis. Other signs taken into consideration included physical and mental alertness, chronic diarrhea and bleeding. A daily dose of 50 mg/kg bw of ceftazidime was administered subcutaneously from the day of initiation of immunosuppression (−2 day) to the last day of treatment (day 14) to prevent any secondary bacterial infection in animals due to immunosuppression. Two days after the 1^st^ dose of immunosuppressants i.e. on the day of infection, the mice were randomly divided into six groups containing 15 animals each. The blood was collected randomly from animals to assure leukopenia (white blood cell count <1000/mm^3^). The leukopenic animals of group II to VI were infected with *A. fumigatus*. Table 1 provides the group wise treatment schedule of the experimental animals.

**Table 1:** Treatment of infected mice with different doses of SCD-1.

Group	Treatment from day 1 to 14
I. Negative control	Sterile water containing 0.5% DMSO, oral
II. Infection control	Sterile water containing 0.5% DMSO, oral
III. Standard drug	Amphotericin B, 1 mg/kg bw, intravenous
IV. Test	SCD-1, 200 mg/kg bw, oral
V. Test	SCD-1, 100 mg/kg bw, oral
VI. Test	SCD-1, 100 mg/kg bw, intraperitoneal

#### Infection

Mice were anesthetized by an intraperitoneal injection of ketamine-xylazine solution (2.5 mg of ketamine and 0.1 g of xylazine/mouse) and then infected intranasally with *A. fumigatus* conidia. Based on the results of a pilot study, a total of 2×10^6^ conidia in 20 µl of PBS were instilled to the animals through nostrils. One hour after infection, 3 mice from each group were euthanized to check conidial delivery to the lungs by counting colony forming units (CFUs) in lungs. The animals of negative control group received 20 µl of PBS through nostrils.

#### Chemotherapy

Treatment of *A. fumigatus* infected mice with SCD-1 was initiated at 24 h post infection (day 1) and was continued upto 14 days (Table 1). The first group (Group I) had control mice that received PBS on the day of infection (day 0) and sterile water containing 0.5% dimethyl sulfoxide (DMSO) from day 1 to day 14. Another control group (Group II) consisted of mice infected with *A. fumigatus* and treated with sterile water containing DMSO only. The infected mice of group III were administered standard drug, Amphotericin B (1 mg/kg bw), intravenously. SCD-1 was dissolved in DMSO and further subdilutions were made in endotoxin free PBS (Sigma Chemicals, USA). The groups (IV–VI) consisted of infected mice receiving 200 mg/kg bw (oral), 100 mg/kg bw (oral) and 100 mg/kg bw (intraperitoneal) of SCD-1, respectively.

### Assessment of antifungal efficacy

The experimental animals were examined daily and the *in vivo* antifungal efficacy of SCD-1 was determined by evaluating the survival of animals, CFU in organs, levels of serum biochemical parameters and histopathology (data not shown). Humane endpoints were used for sacrificing the animals during the course of study. During the study, animals did not show any abnormal signs, pain or distress. As per predetermined protocol, three animals from each group were euthanized on 7^th^ day and remaining animals were euthanized on 14^th^ day by CO_2_ asphyxiation and thereafter cervical dislocation was performed to confirm death. During the study period, all animals were under veterinarian’s supervision and no abnormal behaviour was observed.

#### Survival rate

The animals were monitored daily upto 14^th^ day of infection for signs of moribundity and mortality, if any. The body weight of animals was recorded daily.

#### Quantification of CFU

The mice were kept under constant watch and those getting moribund or survived upto 14 days were euthanized at two different time points (on day 7 and day 14) to determine the fungal burden in different organs. Three animals from each group were euthanized. The lung, liver, kidney and spleen were aseptically removed, cut into small pieces and homogenized in sterile saline containing gentamicin (20 mg/L) and chloramphenicol (400 mg/L) both from Sigma Chemicals, USA. Serial 10-fold dilutions of the homogenate (100 µl) prepared from different tissues, were poured onto SDA plates in triplicate and spread with the help of a glass spreader. The plates were incubated at 37°C for 24 h and then the fungal colonies were counted. The data from two independent experiments was pooled and expressed as number of CFU/g of tissue (mean ± SE). The Epi Info software (ver 3.3.2.) was used to calculate the level of significance using one way analysis of variance.

#### Serum Chemistry

Blood urea nitrogen (BUN), creatinine, serum glutamic pyruvic transaminase (SGPT) and serum glutamic oxaloacetic transaminase (SGOT) were determined using auto-analyser BS-400 (Mindray, China) on day 7 and 14 post treatment.

#### Histopathology

The tissues of lung, liver and kidney were obtained from 3 mice per group on 7^th^ day and were processed for histological examination. The tissue sections were prepared and subjected to stain with hematoxylin and eosin (data not shown).

## Results

### Acute oral toxicity

According to OECD guidelines, pre-defined doses of 2000, 300, 50 and 5 mg/kg bw were used, stepwise, to treat animals in groups having 3 mice each by oral route. Initially, the limit dose of 2000 mg/kg bw of SCD-1 was tested and it was observed that all 3 animals died within 1 h. Although one animal in this group did not show excessive abnormal reaction to the treatment before death, other two showed severe circling movements and convulsions. As per the protocol, subsequently, the next lower dose, i.e. 300 mg/kg bw was administered. All the animals tolerated this dose, therefore, they were observed upto 14 days for adverse symptoms, if any. The animals treated with 300 mg/kg bw of SCD-1 looked like normal and all survived till the end of study period. Post-mortem studies performed on 14^th^ day, did not reveal any internal or external abnormality. Based on the analysis, SCD-1 could be assigned to category IV of the Globally Harmonized System (GHS) for classification of chemicals and its LD_50_ cut-off value was 2000 mg/kg bw. Owing to significant safety level of SCD-1, it was investigated for *in vivo* antifungal efficacy using a mouse model of aspergillosis.

### Antifungal efficacy

**Figure 1:**
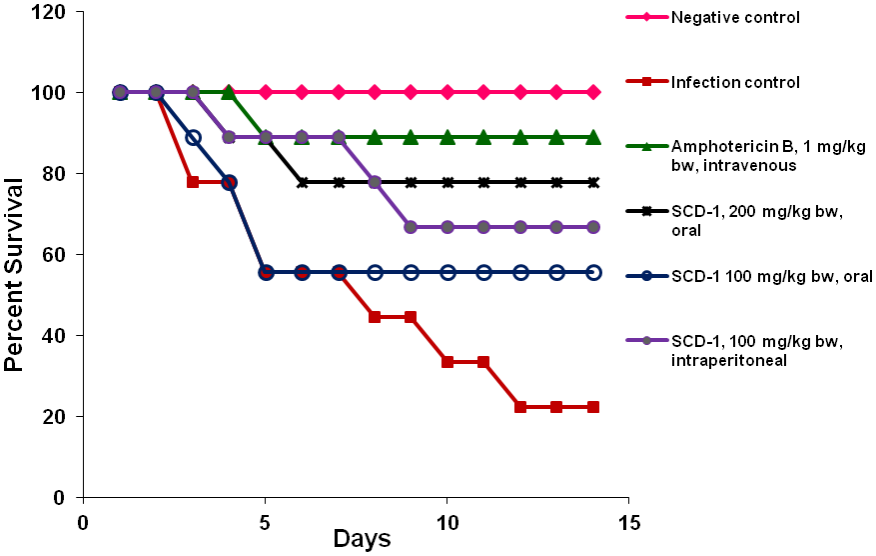
Survival rate in experimental mice. The animals were challenged intranasally with conidia of *A. fumigatus* and treated with different doses of SCD-1, daily, upto 14 days.

The *in vivo* efficacy of SCD-1 against *A. fumigatus*-induced infection was studied in female swiss albino mice. The daily treatment of animals was started 24 h post infection and continued upto 14 days. Results showed that among the animals treated with SCD-1, survival was highest (77.8%) in the group treated with 200 mg/kg bw, orally, followed by the group treated with an intraperitoneal dose of 100 mg/kg bw (66.7%). The animals treated with 1 mg/kg bw of Amphotericin B, intravenously, showed a survival rate of 88.9%. On the other hand, the animals receiving 100 mg/kg bw of SCD-1, orally, exhibited lower survival (55.6%) in comparison to other test groups. The infected-untreated control group showed survival of only 22.3% of animals at the end of 14^th^ day (Figure 1).

**Figure 2:**
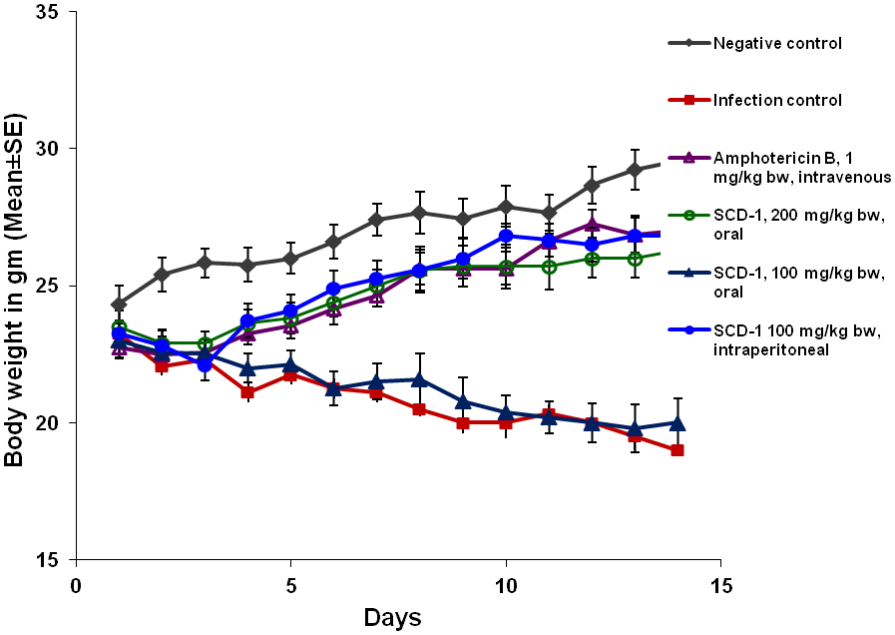
Body weights of experimental mice. The daily weight (Mean ± SE) of animals belonging to different groups was recorded daily over a period of 14 days.

There was a decrease in the body weight of infected animals, which were not given any antifungal treatment. In contrast, uninfected control group that received sterile water containing 0.5% DMSO, orally, showed a weight gain from 24.33±0.68 g (day 1) to 29.55±0.70 g (day 14). The daily oral administration of 200 mg/kg bw of SCD-1 to animals resulted in an increase of body weight from 23.50±0.60 g on day 1 to 26.28±0.60 g on day 14 (Figure 2). This effect was very similar to that observed in Amphotericin B treated group where weight of animals increased from 22.75±0.41 g to 27.00±0.70 g. The animals receiving an intraperitoneal dose of 100 mg/kg bw of SCD-1 also gained weight, in comparison to the infected-untreated animals, as their weight increased from 23.25±0.47 g (day 1) to 26.83±0.43 g (day 14). An oral dose of 100 mg/kg bw of SCD-1 did not aid animals to restore their body weight.

### Fungal burden in organs

**Figure 3:**
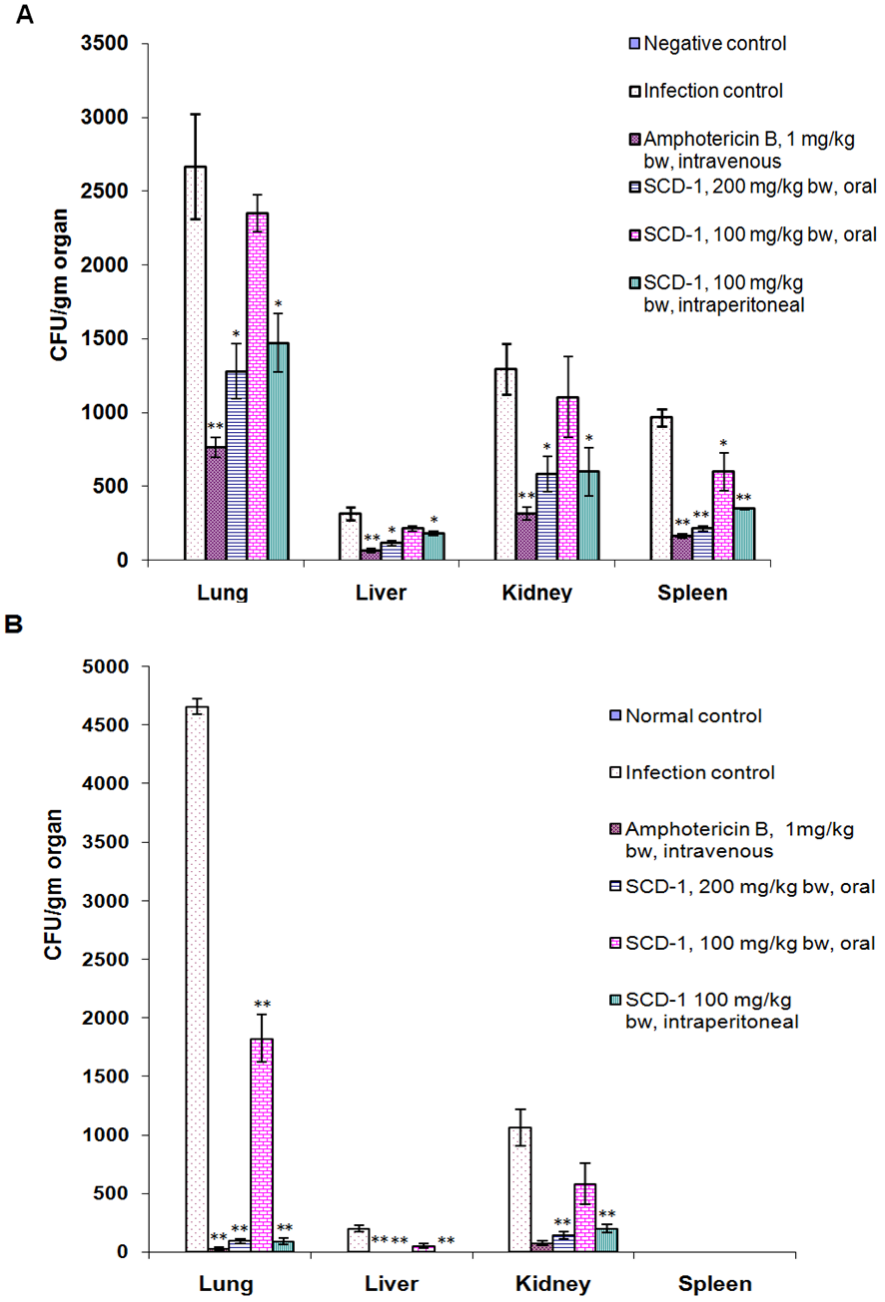
Fungal burden in different organs of experimental animals. The number of CFUs (Mean ± SE) were determined on 7^th^ (A) and 14^th^ (B) day of infection. Asterisks indicate statistically significant differences. *p<0.05, **p<0.001.

The fungal burden in organs of experimental animals was determined by calculating the CFUs in tissue homogenates of organs at 7^th^ and 14^th^ day. Figure 3 shows the number of CFUs of *A. fumigatus* in different organs of mice. Lung being the primary target, was found to be most susceptible organ to *A. fumigatus* infection. The animals of the infected-untreated control group developed 2666±356 CFU/g in lung on day 7 after infection, which increased to 4659±68 on day 14 (Figure 3A and B). On 7^th^ day, fungal colony count was reduced (*p*<0.05) to 1280±185 and 1472.72±197.5 (*p*<0.05) in lungs of mice treated with SCD-1 by oral and intraperitoneal doses of 200 and 100 mg/kg bw. The CFU in lung of mice treated with 100 mg/kg bw of SCD-1 (orally) was reduced (*p*>0.05) to 2348.48±124.0 from 2666±356. The number of CFU/g in liver, kidney and spleen of infected-untreated control animals on 7^th^ day was 316.66±44.0, 1295.45±171.0 and 966±60, respectively. There was a significant decrease (*p*<0.05) in number of CFUs in liver and kidney of animals which were treated with 200 mg/kg bw, (oral) and 100 mg/kg bw (intraperitoneal) of SCD-1. In spleen, there was a highly significant (*p*<0.001) reduction with oral and intraperitoneal doses of 200 and 100 mg/kg bw, of SCD-1. In addition, the splenic colony counts were reduced significantly after oral treatment with 100 mg/kg bw of SCD-1. The treatment with Amphotericin B led to a marked decrease (*p*<0.001) in the number of fungal colonies in all organs on 7^th^ day of infection.

On 14^th^ day after infection, there were 4659.09±68.18, 200.00±25.0 and 1068.18±159.09 colonies in lung, liver and kidney of the infected-untreated animals. The SCD-1 treatment resulted in highly significant reduction (*p*<0.001) in fungal colony count in lungs of animals belonging to all the three groups treated with SCD-1. Similarly, significant removal (*p*<0.001) of the pathogen from kidney was observed in the groups treated with 200 mg/kg of SCD-1 (oral) and 100 mg/kg bw by intraperitoneal route. It was interesting to note that the infection was almost completely resolved from liver and spleen of animals treated with SCD-1 (Figure 3B).

### Serum Chemistry

The concentration of serum biochemical parameters was determined on 7^th^ and 14^th^ day of infection (Table 2). On day 7, the levels of BUN, creatinine, SGPT and SGOT in uninfected control animals were found to be 19.83±0.28 mg/dl, 0.43±0.03 mg/dl, 161.00±11.27 U/litre and 313.67±8.09 U/litre, respectively. The *A. fumigatus* infection resulted in a significant rise (*p*<0.05) in the levels of these biochemical parameters in infected-untreated animals as compared to the negative control group. The treatment with 200 mg/kg bw, orally and 100 mg/kg bw, intraperitoneally, of SCD-1 led to a significant reduction (*p*<0.05) in the levels of SGPT and SGOT. An intraperitoneal dose of 100 mg/kg bw caused a significant reduction (*p*<0.05) in the level of BUN. A significant decrease in level of creatinine was achieved by an oral dose of 200 mg/kg bw. The *A. fumigatus* infection mediated increase (*p*<0.05) in level of these molecules was observed on day 14 also, which was brought down significantly (*p*<0.05) by the treatment with 200 mg/kg bw, oral and 100 mg/kg bw, intraperitoneal doses of SCD-1 (Table 2). An oral dose of 100 mg/kg bw of SCD-1 did not result in significant (*p*<0.05) reduction in the level of serum biochemical parameters post fungal infection.

**Table 2:** The effect of SCD-1 treatment on serum biochemical parameters in mice infected with *A. fumigatus* a.

Day	Group	BUN (mg/dl)	Creatinine (mg/dl)	SGOT (U/L)	SGPT (U/L)
7.	I	19.83±0.28	0.43±0.03	313.67±8.09	161.00±11.27
	II	22.20±0.23^b^	0.67±0.07^b^	385.00±5.13^b^	204.67±5.46^b^
	III	17.23±2.61	0.40±0.06^c^	261.67±14.3^c^	86.67±1.76^c^
	IV	18.40±1.91	0.37±0.03^c^	315.67±9.60^c^	126.67±9.61^c^
	V	22.57±0.39	0.53±0.03	359.33±30.56	139.67±10.74
	VI	20.47±0.47^c^	0.50±0.06	339.67±9.35^c^	157.00±2.03^c^
14.	I	15.67±0.60	0.43±0.03	280.67±12.67	127.33±8.03
	II	24.25±2.05^b^	0.75±0.15^b^	470.00±15.00^b^	171.50±7.50^b^
	III	17.73±1.19^c^	0.43±0.03^c^	253.00±20.43^c^	69.67±4.63^c^
	IV	19.37±0.69^c^	0.43±0.03^c^	266.33±21.31^c^	106.67±7.22^c^
	V	21.03±1.29	0.53±0.03	375.00±51.93	196.33±9.91
	VI	20.17±1.07^c^	0.43±0.03^c^	297.00±29.74^c^	136.33±20.17^c^

a The value are expressed as mean ± SE.

b *p*<0.05, compared with the values of negative control mice.

c *p*<0.05 compared with the values of infection control mice.

### Histopathology

The haematoxylin and eosin stained sections of lung, liver and kidney were examined on 7^th^ day. The sections from negative control animals did not show any signs of abnormality or any lesions (data not shown). In the lungs of infected control animals, there was extensive necrosis with multifocal fungal granulomatous inflammatory foci having large number of dichotomously branched growing hyphae in the tissue. The treatment of infected animals, orally, with 200 mg/kg bw of SCD-1 markedly reduced lesions with only mild inflammation in lungs. The liver and kidney of infected-untreated animals showed marked diffused granular degeneration. After treatment with SCD-1, the inflammatory infiltrates in these organs were reduced significantly (data not shown).

## Discussion

In spite of increasing mortality, therapeutic options for the treatment of aspergillosis have been limited to few classes of antifungals [27], [28]. Therefore, it is imperative to develop and characterize new molecules with no or minimal side effects for the effective management of disease. In an effort to explore potent anti-*Aspergillus* molecules, present study was aimed to examine the safety and antifungal efficacy of SCD-1, a synthetic coumarin derivative using mouse model. Although coumarins have been explored for their biological properties such as anti-inflammatory, cytotoxic, anti-tubercular and angiogenic [19]–[22] etc, studies focusing on their antifungal efficacy are limited in number, only *in vitro* antifungal activity has been indicated in some reports [29], [30]. Till date, there have been no reports on the toxicity and *in vivo* antifungal activity of coumarins. We have previously reported the *in vitro* antifungal activity of SCD-1 [23], and its impact on the proteomic machinery of *A. fumigatus* [24]. The present study addressed for the first time the *in vivo* safety and antifungal efficacy of a synthetic coumarin derivative.

The analysis of safety of a compound is a crucial step in determining its biological property. The adoption of OECD guidelines for acute toxicity testing has been an important landmark in animal welfare. This method is based on biometric evaluations with fixed doses of the compound to be tested. The doses are adequately separated to enable a substance to be classified according to Globally Harmonised System for the classification of chemicals. Accordingly, the starting dose level should be that which is most likely to produce mortality in some of the dosed animals. Therefore, for the present study the starting dose was decided to be 2000 mg/kg body weight. Based on the results, downstream methodology of OECD was followed. The oral acute toxic class method (OECD) was developed as an alternative to replace the oral LD_50_ test. It is a stepwise procedure that does not intend to allow calculation of an exact LD_50_ for a substance, but does allow determination of defined concentration ranges where lethality may be expected. By utilising this approach, sufficient information is obtained on the acute toxicity of the test substance to enable its classification simultaneously reducing the number of animals used for testing [25], [31]. Based on the results of toxicity experiments using two doses i.e 300 and 2000 mg/kg bw, the guidelines allowed extrapolation of data and the LD_50_ cut-off value could be determined. The LD_50_ cut-off value of SCD-1 was established to be 2000 mg/kg bw. It was, concluded that SCD-1 for treating aspergillosis in animals was quite safe. The compound was classified into category IV of GHS, this system of classification was developed to increase the consistency among experimental set-ups in different nations [32]. Seidle *et al* [33] emphasised that in acute toxicity studies, the classification and labelling of substances is most important.

SCD-1 demonstrated protection against infection by *A. fumigatus* in an intranasal murine model of aspergillosis. The administration of *A. fumigatus* through intranasal route has been reported to mimic the natural sinopulmonary route of infection in humans [34]. The animals were observed up to 14 days [35]–[37]. To prevent unnecessary discomfort, all the animals were euthanized by the 14^th^ day, as the weight of infected-untreated animals decreased to approximately 20% of the initial body weight. It was observed that mice without any antifungal treatment showed 22.3% survival whereas the animals receiving 200 mg/kg bw of SCD-1, orally, showed a survival rate of 77.8%. The group treated with 100 mg/kg bw by oral and intraperitoneal routes were also effective in increasing survival of animals. A higher dose of SCD-1 by intraperitoneal route was not tested considering that a compound should be effective at lower concentration via intraperitoneal route in comparison to the oral route. The animals treated with 1 mg/kg bw of standard drug, Amphotericin B by intravenous route showed a survival of 88.9%. Despite its high efficacy, the clinical application of Amphotericin B is often limited by its dose dependent nephrotoxicity [38]. The administration of Amphotericin B by intravenous route and its low oral bioavailability are other major concerns [39]. In mice, the LD_50_ of Amphotericin B by intravenous route has been reported to be 2–3 mg/kg bw [40]. The major finding of this experiment was the comparable antifungal effect of SCD-1 to the standard drug, Amphotericin B, in terms of increased survival and decreased fungal burden.

The infected-untreated animals demonstrated a decrease in body weight as compared to the uninfected control group. In contrast, the animals receiving SCD-1 by oral and intraperitoneal routes showed an increase in body weight comparable to the uninfected control animals. It could thus be inferred that the weight loss in infected animals was infection-mediated and the doses of SCD-1 which controlled fungemia resulted in recovery from the weight loss.

Besides the survival rate of animals as a parameter for therapeutic efficacy of SCD-1, *A. fumigatus* colony counts were calculated to determine the fungal burden. Several researchers have employed CFU method to quantitate the fungal burden in the tissues and the efficacy of antifungal drugs. Singh *et al* [41] reported CFU method and quantitative polymerase chain reaction to be equally useful for determining the efficacy of caspofungin. We observed that lungs were most heavily infected and primary target of infection [42], this was expected as the fungus was administered through the intranasal route. Lewis *et al* [43] showed reduction in lung CFUs after treatment with Amphotericin B in mice infected intranasally with *A. fumigatus* and sacrificed after 4 days of infection. We also observed a significant decrease in lung colony counts in animals on 7^th^ and 14^th^ day of infection after treatment. SCD-1 was observed to control fungemia in other organs also. The fungal infection was cleared by 14^th^ day from liver of the animals in most of the treated groups. Also, the pathogen was cleared from the spleen of animals belonging to all groups, with or without antifungal treatment. Sionov *et al* [38] determined the efficacy of combination therapy using Amphotericin B or Amphotericin B intralipid mixture and caspofungin in mice infected with *A. fumigatus* by intravenous route. The authors reported complete removal of pathogen from spleen and kidney of animals belonging to treatment groups on 14^th^ day [44].

A significant decrease in the level of serum biochemical parameters (BUN, creatinine, SGPT and SGOT) after treatment with SCD-1, in infected animals, suggested protective effect of compound on vital organs. Wallace *et al* (1997) determined the *in vivo* antifungal efficacy of liposomal nystatin in a neutropenic model of disseminated aspergillosis and observed no significant difference in the level of BUN and creatinine on day 5 or 19 as compared to their pre-dose level [45].

The histopathological observations also showed that there was no or minimal inflammation and necrosis in lungs, liver and kidney of the animals treated with SCD-1, indicating its effectiveness in protecting the important organs of the body.

In conclusion, present study was focused on *in vivo* safety and antifungal efficacy evaluation of a synthetic coumarin, SCD-1. It was found to be highly safe to the experimental animals, upto a dose of 300 mg/kg bw. The SCD-1 had protective effect against *A. fumigatus* infection as the treatment resulted in increased survival and effective pathogen clearance from organs by reducing the fungemia in animals. The most important aspect was the manifold safety of SCD-1 as demonstrated by its high LD_50_ cut-off value (2000 mg/kg bw). In this study, we have tested three different doses of SCD-1 by different routes but much higher doses of SCD-1 can be administered safely owing to its high tolerable dose, thereby, resulting in an improved therapeutic profile. Further studies are warranted to establish the utility of SCD-1 in treatment of IA as it presents a lead for development of potent antifungal therapies.

## Supporting Information

Checklist S1 **The ARRIVE Guidelines Checklist.** The ARRIVE guidelines were followed for reporting this study.
(DOC)
